# The Identification of Cable Bacteria Attached to the Anode of a Benthic Microbial Fuel Cell: Evidence of Long Distance Extracellular Electron Transport to Electrodes

**DOI:** 10.3389/fmicb.2017.02055

**Published:** 2017-10-24

**Authors:** Clare E. Reimers, Cheng Li, Michael F. Graw, Paul S. Schrader, Michael Wolf

**Affiliations:** ^1^College of Earth, Ocean and Atmospheric Sciences, Oregon State University, Corvallis, OR, United States; ^2^Teledyne Benthos, North Falmouth, MA, United States

**Keywords:** cable bacteria, microbial fuel cell, sulfur oxidation, filaments, pili, extracellular electron transfer, anaerobic respiration

## Abstract

Multicellular, filamentous, sulfur-oxidizing bacteria, known as cable bacteria, were discovered attached to fibers of a carbon brush electrode serving as an anode of a benthic microbial fuel cell (BMFC). The BMFC had been operated in a temperate estuarine environment for over a year before collecting anode samples for scanning electron microscopy and phylogenetic analyses. Individual filaments were attached by single terminus cells with networks of pilus-like nano-filaments radiating out from these cells, across the anode fiber surface, and between adjacent attachment locations. Current harvesting by the BMFC poised the anode at potentials of ~170–250 mV vs. SHE, and these surface potentials appear to have allowed the cable bacteria to use the anode as an electron acceptor in a completely anaerobic environment. A combination of catalyzed reporter deposition fluorescent *in situ* hybridization (CARD-FISH) and 16S rRNA gene sequence analysis confirmed the phylogeny of the cable bacteria and showed that filaments often occurred in bundles and in close association with members of the genera *Desulfuromonas*. However, the *Desulfobulbaceae* Operational Taxonomic Units (OTUs) from the 16S sequencing did not cluster closely with other putative cable bacteria sequences suggesting that the taxonomic delineation of cable bacteria is far from complete.

## Introduction

Investigations of mechanisms that divert electrons from microbial catabolic activity to generate electricity between electrodes of a microbial fuel cell (MFC) have been instrumental in recent discoveries of new respiratory reactions, microbial syntrophy, and the conductive properties of biofilms (Schröder, 2007; Nevin et al., 2009; Lovley, 2012, 2017; Malvankar et al., 2012, 2014; Li et al., 2016). Electron sharing between bacteria and an electrode can mimic how cells transfer electrons directly or through electron shuttles (e.g., flavins) to either other cells, or to solid-phase minerals or humic substances. There is now a diversity of microorganisms known to employ various means of extracellular electron transfer such as constructing electrically conductive pili, producing outer membrane *c*-type cytochromes, excreting redox shuttling compounds that bind to surfaces, and connecting through conductive abiotic materials (Gorby et al., 2006; Marsili et al., 2008; Summers et al., 2010; Kato et al., 2012; Lovley, 2012). The most intensively studied are *Geobacter* and *Shewanella* species, many strains of which originate from the marine environment or riverine sediments, where they commonly reduce iron and manganese (oxyhydr)oxide minerals or elemental sulfur (S°) (Lovley et al., 2011; Pirbadian et al., 2014).

Chemical reactions that precipitate metal (oxyhydr)oxides and S° often coincide with a redox potential gradient between oxic and anoxic conditions (Berner, 1963; Stumm and Morgan, 1981). In coastal marine sediments that receive high inputs of reduced carbon as particulate organic matter, dissimilatory sulfate reduction can be the main pathway of organic carbon oxidation leading to the buildup of dissolved sulfide (ΣH_2_S = H_2_S + HS^−^+ S^2−^) at depths that may be just a few mm or cm below an oxic sediment-water interface. Significant quantities of the dissolved sulfide react chemically with iron (oxydr)oxides to form FeS, S°, and FeS_2_, while a fraction is typically reoxidized after transport by diffusion or bioturbation to overlying oxic zones (Jørgensen and Kasten, 2006).

Recently, a novel type of sulfide oxidation was documented in marine sediments whereby filamentous cable bacteria affiliated with the deltaproteobacterial family *Desulfobulbaceae* mediate intra-species cell-to-cell electron transport over mm to cm-scale distances that coincide with suboxic zones (Nielsen et al., 2010; Pfeffer et al., 2012; Nielsen and Risgaard-Petersen, 2015; Vasquez-Cardenas et al., 2015; Trojan et al., 2016). Substrate utilization experiments and chemical evidence indicate these cable bacteria are motile chemoorganotrophs that readily harvest electrons from sulfide at depth, passing the electrons along a chain of redox sites to oxygen or nitrate in the first millimeters of coastal sediments (Malkin et al., 2014; Marzocchi et al., 2014; Malkin and Meysman, 2015; Vasquez-Cardenas et al., 2015). However, laboratory efforts to connect cable bacteria to electrodes or to cultivate them in axenic culture have not been successful (Nielsen and Risgaard-Petersen, 2015; Trojan et al., 2016; Lovley, 2017). This has left unresolved their exact mechanism for conducting electrons, the involvement of internal vs. external redox couples, and whether cable bacteria can transfer electrons beyond the cable terminus to solid extracellular substrates such as iron-oxide minerals.

As serendipity has often led to discoveries of unknown microbial processes and associations, we were excited to observe by scanning electron microscopy (SEM) cable bacteria filaments firmly attached by their terminus cells to carbon fibers of a benthic microbial fuel cell (BMFC) anode after it had harvested electricity in the marine environment for over 1 year. Subsequent catalyzed reporter deposition fluorescent *in situ* hybridization (CARD-FISH) and 16S rRNA gene sequence analysis confirmed the phylogeny as *Desulfobulbaceae*, and the BMFC performance history indicates that these bacteria were using the anode as an electron acceptor after being deprived of access to oxygen for many months. In the attached mode, the cable bacteria co-occur with nano-filament networks and other electrogenic bacteria, especially those belonging to the *Desulfuromonadaceae* family. The evidence for these assertions and their possible implications for understanding the mechanisms for long-range electron transfer through cable bacteria are the focal points of this research article. We also re-evaluate representations of the microbial metabolic networks that may catalyze electron transfer to marine BMFCs.

## Materials and methods

### BMFC design

**Figure 1:**
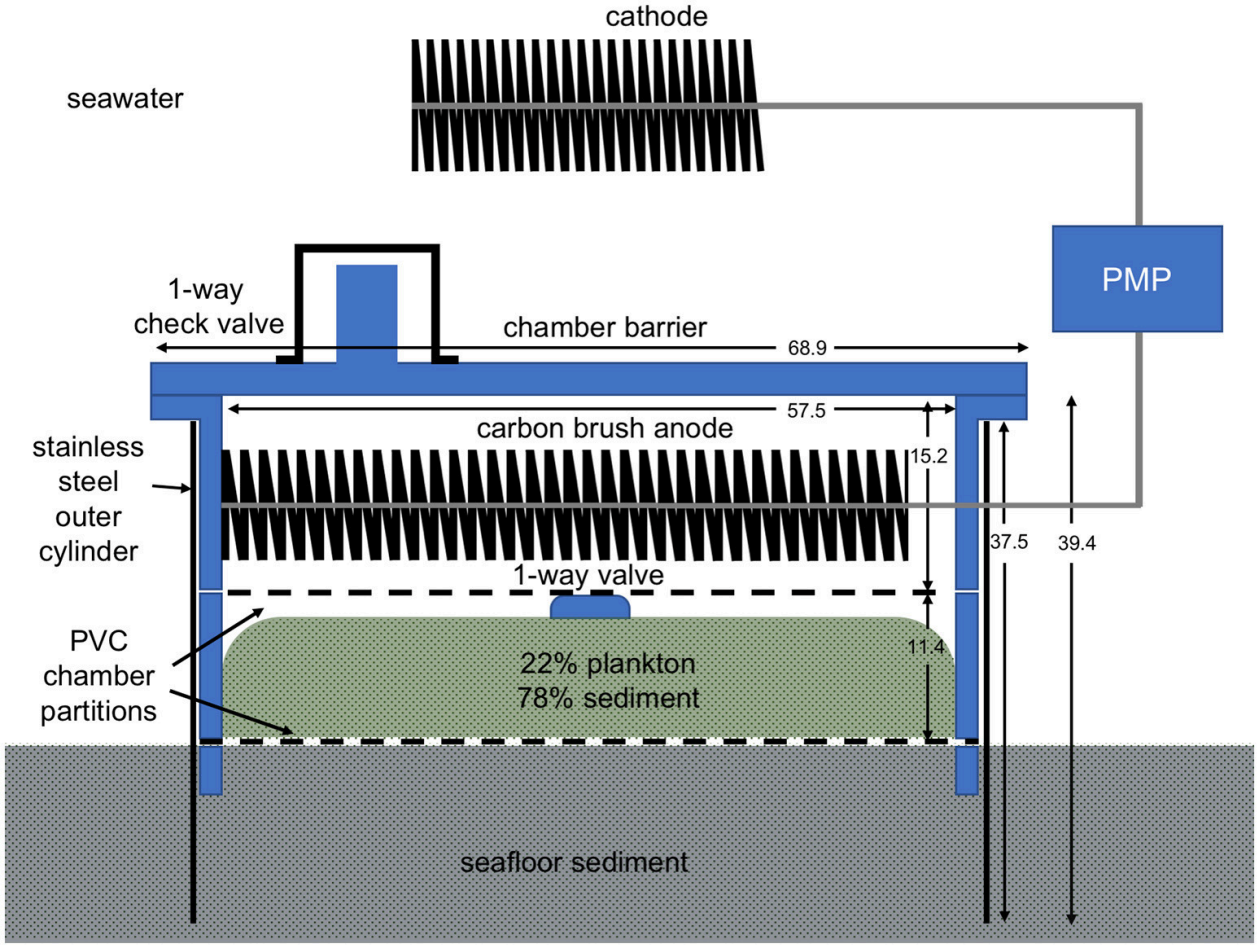
Schematic of the BMFC design used in this study. Dimensions are in cm. Microbial respiration creates an anoxic environment inside the anode chamber.

This study transpired after the deployment of a BMFC in an estuarine environment. As in previous developmental designs, the BMFC relied on a benthic chamber (footprint = 0.259 m^2^, open at the bottom for insertion into the seafloor) to isolate an anode from dissolved oxygen, while the cathode was exposed to oxic seawater in a turbulent environment (Gong et al., 2011; Schrader et al., 2016; Figure 1). To help fuel the BMFC, an organic-rich sediment layer was preloaded in the anode chamber between vertically-separated, perforated, polyvinyl chloride (PVC) dividers. This layer was prepared by mixing 2.7 kg dried plankton with 9.5 kg natural wet sediment from the study site. The plankton source was Plankton Gold Flake (BrineShrimpDirect, Ogden, Utah) that the distributor reports contains 45% protein, 12% fat, 6% ash, and 6% moisture. The mixture was spread onto a layer of cheesecloth that was cut to fit the bottom perforated PVC divider, situated below the anode. The chamber was also fitted with a rolled stainless-steel outer shroud to help penetrate the seafloor to the depth of the lower PVC divider.

Anode and cathode were formed from 4- to 2-m long “carbon brushes” produced with Panex 35 carbon fibers (diameters = 5–10 μm, lengths = 10.2 cm) intertwined mid-length in #11 titanium stem wire (400,000 fibers per 2.54 cm; Mill-Rose). Individual carbon fibers are cylindrical providing a high surface area for bioelectrochemical reactions. The anode brush was coiled on top of the upper PVC divider in the chamber. The cathode was supported above the chamber as described below.

### BMFC operation

**Figure 2:**
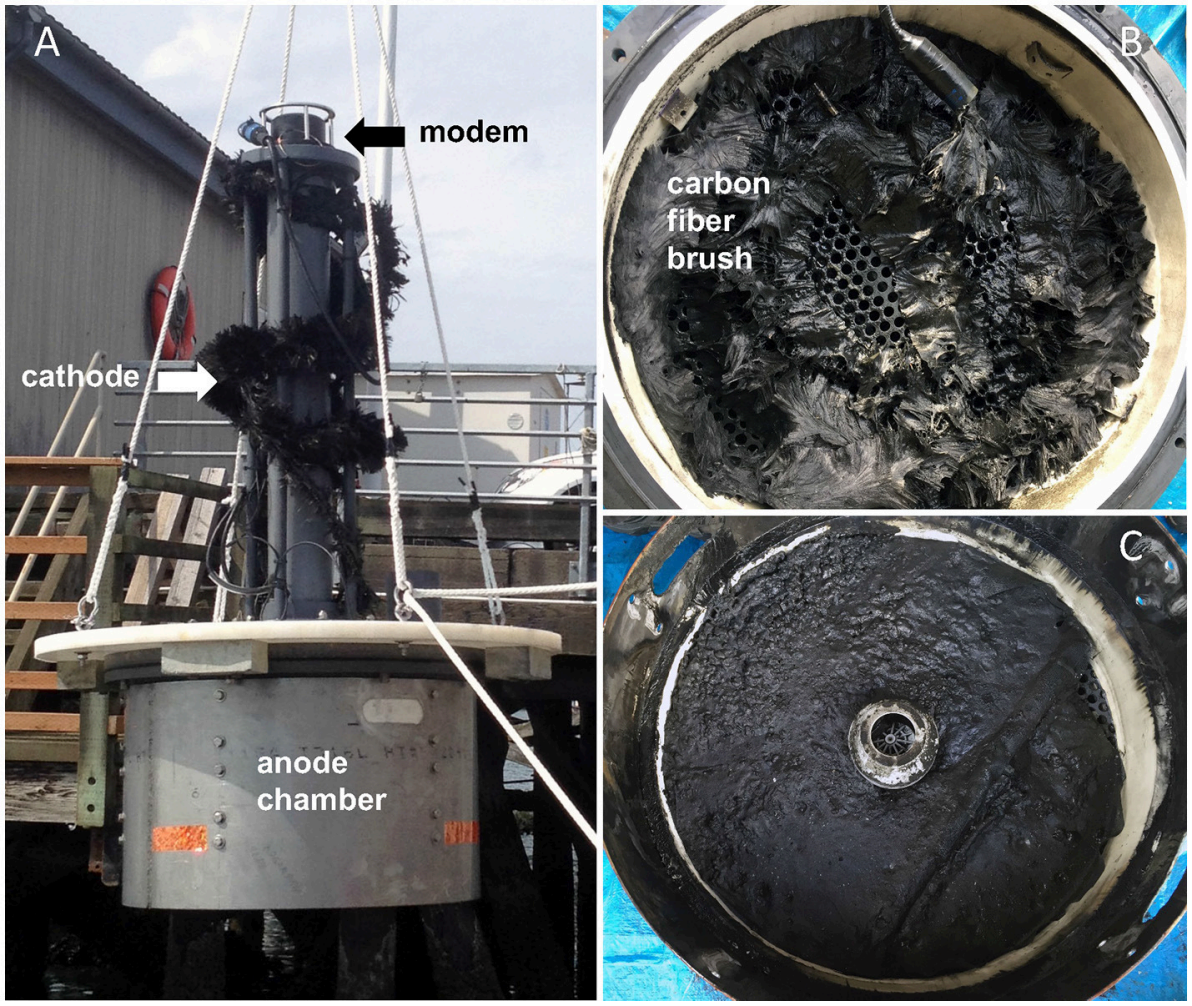
**(A)** Image of the benthic microbial fuel cell (BMFC) being launched into Yaquina Bay, Oregon. The stainless shroud around the anode chamber is visible at the base. The cathode is wound around a PVC pipe supporting the acoustic modem. **(B)** Appearance of the anode carbon fiber brush after recovery and removing the lid of the anode chamber. **(C)** Layer of anoxic plankton-enriched sediment from inside the anode chamber after recovery.

The BMFC operation was controlled by a power management platform (PMP) (Reimers, 2015; Schrader et al., 2016) with customized firmware. Electricity harvested by the BMFC was routed to two stacks of 3.8 V Li-ion cells connected in series which created an energy storage capacity of 57 Wh. Power for the PMP microprocessor was drawn from a supercapacitor (2 V) that acted as a mid-point for a two-stage charge pump circuit to step-up voltage and pass charge to the Li-ion cells. The Li-ion batteries served to power an integrated optode sensor (Aanderaa model 4,330) that measured dissolved oxygen and temperature at programmable intervals ranging from every 1 h to every 10 min, and an acoustic modem (Teledyne Benthos 920 Series ATM-925) used for data storage, data recovery, and sending control commands to the PMP. The modem's power requirements varied from 2.5 mW in hibernate mode, to 0.4 W when awakened to listening mode, to 0.8–2 W in transmit mode (depending on the transmit power level). The modem was mounted in a PVC column centered above the anode chamber, with the cathode wrapped around the column (Figure 2A).

The BMFC was deployed within Yaquina Bay, Oregon on September 16, 2014 at a location of previous experiments near the Oregon State University dock (Latitude 44° 37′31 N, Longitude 124° 2′41 W, MLW depth 7 m; Ryckelynck et al., 2005; Nielsen et al., 2007; Gong et al., 2011). Yaquina Bay is a small drowned river estuary dominated by strong tidal exchange with the coastal ocean (Brown and Ozretich, 2009). A storm overturned the BMFC chamber on deployment day 40, but divers righted it and installed sand anchors (day 55) to ensure platform stability during the remainder of the deployment. The water temperature and current speed at the site were observed to typically average 12.0 ± 0.7°C and 6.3 ± 7.0 cm s^−1^ (based on near bottom SonTek Argonaut Acoustic Doppler Velocimeter measurements made adjacent to the BMFC during deployment days 91–135). The BMFC operated continuously for a total of 412 days until recovered using a shore-side crane to pull it from the seafloor and out of the water. Logged data included sensor readings (dissolved oxygen and temperature), and energy harvesting parameters that included hourly records of the cell voltage of the BMFC (i.e., cathode vs. anode potential), energy out of the BMFC, and the voltages of the rechargeable Li-ion batteries. BMFC performance was assessed further by conducting a polarization (Logan et al., 2006) daily whereby the BMFC was isolated and cell voltage measured after allowing current to flow across each of nine resistances (open circuit, 100, 75, 50, 35, 25, 15, 10, and 3.25 ohms) for 6 min. After each complete polarization, the cell potential of the BMFC was adjusted by the PMP to a sustainable operating voltage derived as:

$$\begin{array}{l} {\text{V}}_{\text{operating}}={\text{V}}_{\text{max}}+0.25^{*}\left({\text{V}}_{\text{oc}}-{\text{V}}_{\text{max}}\right) \end{array}$$

where V_max_ represents the polarization voltage of maximum power production and V_oc_ was the observed open circuit voltage. However, when the Li-ion storage cells were fully charged (≥3.89 V) the PMP did relax energy harvesting, allowing the BMFC to approach open circuit. This condition occurred periodically 80–300 days after initial deployment when large energy demands were not being placed on the BMFC.

Over the last ~110 days of the experiment, more frequent, and then by the last 75 days more prolonged, modem transmissions were conducted purposely to increase the energy drawn from the Li-ion cells through the PMP and from the BMFC. Consequently, during the last 75 days the average V_operating_ declined and current was drawn continuously (except for 6 min of open circuit at the start of each daily polarization cycle). Current densities were calculated as the measured current normalized to the footprint area inside the chamber (0.259 m^2^).

### Anode fiber and sediment sampling

Two hours after hoisting the BMFC from its deployment location, the anode chamber was opened to expose the anode and internal sediment (Figures 2B,C). Clippings of carbon fibers were collected from 10 locations spaced roughly evenly along the 4 m-long coil of anode using alcohol-sterilized shears and forceps. Splits of these samples were placed in whirl-top bags and immediately transferred to a freezer (−50°C). Other splits were transferred to a fixative (1.25% glutaraldehyde and 1.3% osmium tetraoxide, after Ishii et al., 2008) and retained for microscopy.

The artificially enriched internal sediment within the anode chamber appeared black and gelatinous after recovery. After exposure, a ~50 cm^3^ sample was transferred to a beaker, and whole sediment pH measured at 15°C with a glass combination electrode. The pH electrode was calibrated using National Bureau of Standards (NBS) buffer standards before insertion into the sediment. Additional samples of internal sediment were transferred to 50 cm^3^ centrifuge tubes and centrifuged at 3,800 rpm for 10 min at 15°C. Separated pore waters were then vacuum filtered through 1.2 μm glass fiber filters (GF/C), and the filtrate frozen for later nutrient analyses by autoanalyzer. The solids in each centrifuge tube were also frozen. Subsamples of the sediment solids were later dried, ground and analyzed for organic C and N contents after carbonates were removed by concentrated HCl fumigation. These analyses were made using a NC2500 ThermoQuest Elemental Analyzer with acetanilide as the calibration standard (modified from Verardo et al., 1990).

### Light microscopy and SEM

Small clusters of carbon fibers from the BMFC anode that had been stored in fixative were observed under bright field microscopy at 100x, a few days after recovery, as an exploratory procedure. In these initial images, filamentous structures were visible and often appeared to span between and across fibers (Figure S1) prompting further investigation. Later, other fiber clusters from the 10 sampling locations were dehydrated in a graded series of ethanol from 10 to 100%. Specimens were mounted on aluminum SEM stubs with double-sided carbon tape, critical-point dried using an EMS 850 Critical Point Dryer, and sputter-coated with gold and palladium using a Cressington 108 sputter coater. The resultant specimens were observed under a FEI Quanta 600FEG ESEM at 5–10 kV. This instrument also provided elemental spectra by X-Ray Energy Dispersive Spectrometry (EDS).

### CARD-FISH

To verify that the multi-cellular filaments observed as attached to carbon fibers by SEM were cable bacteria, anode carbon fiber samples that had been frozen were examined by catalyzed reporter deposition-fluorescence *in situ* hybridization (CARD-FISH) using a *Desulfobulbaceae*-specific oligonucleotide probe (DSB706; 5′-ACC CGT ATT CCT CCC GAT-3′) modified with horseradish peroxidase (Pernthaler et al., 2002; Lücker et al., 2007). Samples were prepared according to the procedures suggested by previous studies (Wendeberg, 2010; Malkin et al., 2014). Frozen carbon fiber samples were thawed, fixed in 2% formaldehyde for 10 h at 4°C, and then stored in 50% ethanol at −20°C. A small section of the fixed sample of carbon fibers (0.25 × 0.25 cm) was cut out, gently rinsed by Millipore water, and embedded on glass slides by using 0.1% agarose gel. Embedded samples were allowed to air-dry and then sequentially permeabilized by 10 mg/mL of lysosome (2 h at 37°C) and achromopeptidase (1 h at 37°C). After permeablization, endogenous peroxidases were inactivated by incubation in H_2_O_2_ (0.15% in methanol) for 30 mins at room temperature (~25°C). The hybridization process was performed in standard hybridization buffer at 46°C with 45% formamide for 7 h. Catalyzed reporter deposition was performed by using Alexa Fluro 488 tyramides reagent (ThermoFisher, Waltham, MA, United States). 4′,6-diamidino-2-phenylindole (DAPI) was applied as a counter stain in some treatments. Hybridization samples were visualized using confocal laser scanning microscopy (LSM 780, Zeiss, Jena, Germany).

To investigate the potential synergistic relationship on carbon fibers, multicolor CARD-FISH was performed on a few samples by using an additional *Desulfuromonadales*-specific oligonucleotide probe (DRM432; 5′- CTT CCC CTC TGA CAG AGC−3′) modified with horseradish peroxidase. To perform the multicolor CARD-FISH, samples were treated with 0.15% H_2_O_2_ after the first catalyzed reporter deposition to inactivate the horseradish peroxidase from the first hybridization. The inactivated samples were then hybridized with the DRM 432 probe in standard hybridization buffer at 46°C with 40% formamide for 4 h and sequentially stained by a secondary catalyzed reporter deposition using Alexa Fluro 555 tyramides reagent.

Three types of controls were performed and visualized: (1) sterile carbon fibers from an unused carbon brush, (2) anode carbon fibers from the deployed BMFC that went through all procedures except the oligonucleotide probe was omitted during the hybridization step, and (3) anode fibers from the deployed BMFC that did not receive any of the hybridization and catalyzed reporter deposition processing (Figure S2). It is noteworthy that the samples had been stored frozen and unfixed for more than a year before processing for CARD-FISH.

### Microbial community characterizations

Total DNA was extracted from three anode carbon fiber samples, from a sample of sediment from inside the anode chamber, and from sediment collected by a diver next to the BMFC at the study site. DNA extraction, amplification, and sequencing (Illumina MiSeq Reagent Kit v3, 2 × 300 bp) was performed by the Research and Testing Laboratory (Lubbock, TX). DNA was extracted using a MoBio PowerSoil DNA Extraction Kit with a bead beating step, and bacterial and archaeal 16S rRNA genes were amplified by PCR with primers 357wF (5′-CCTACGGGNGGCWGCAG-3′) and 785R (5′-GACTACHVGGGTATCTAATCC-3′).

Sequences were processed using mothur (v.1.36.1) as described previously (Kozich et al., 2013). Sequences were aligned to the Silva SSU Ref NR database (v.119) and clustered into operational taxonomic units (OTUs) at 97% similarity. Singleton OTUs were removed and samples were rarefied to 15,712 sequences using 100 random iterations prior to analysis. The *vegan* package for R (Oksanen et al., 2013) and Primer 6 with the Permanova+ add-on (Gorley and Clarke, 2006) were used for statistical analysis and ordination.

Representative sequences for the 45 most abundant OTUs classified to the family *Desulfobulbaceae* were extracted and aligned to 16S sequences from previously identified cable bacteria (Schauer et al., 2014; Trojan et al., 2016) and previous BMFC field studies (Holmes et al., 2004; Nielsen et al., 2008) or lab studies (Ishii et al., 2013b) using ClustalW. A phylogenetic tree was constructed using RaxML with 1,000 bootstraps.

Sequences from this study were deposited to Genbank's Sequence Read Archive under accession PRJNA388503.

## Results

### Microbial fuel cell performance and anode environmental conditions

**Figure 3:**
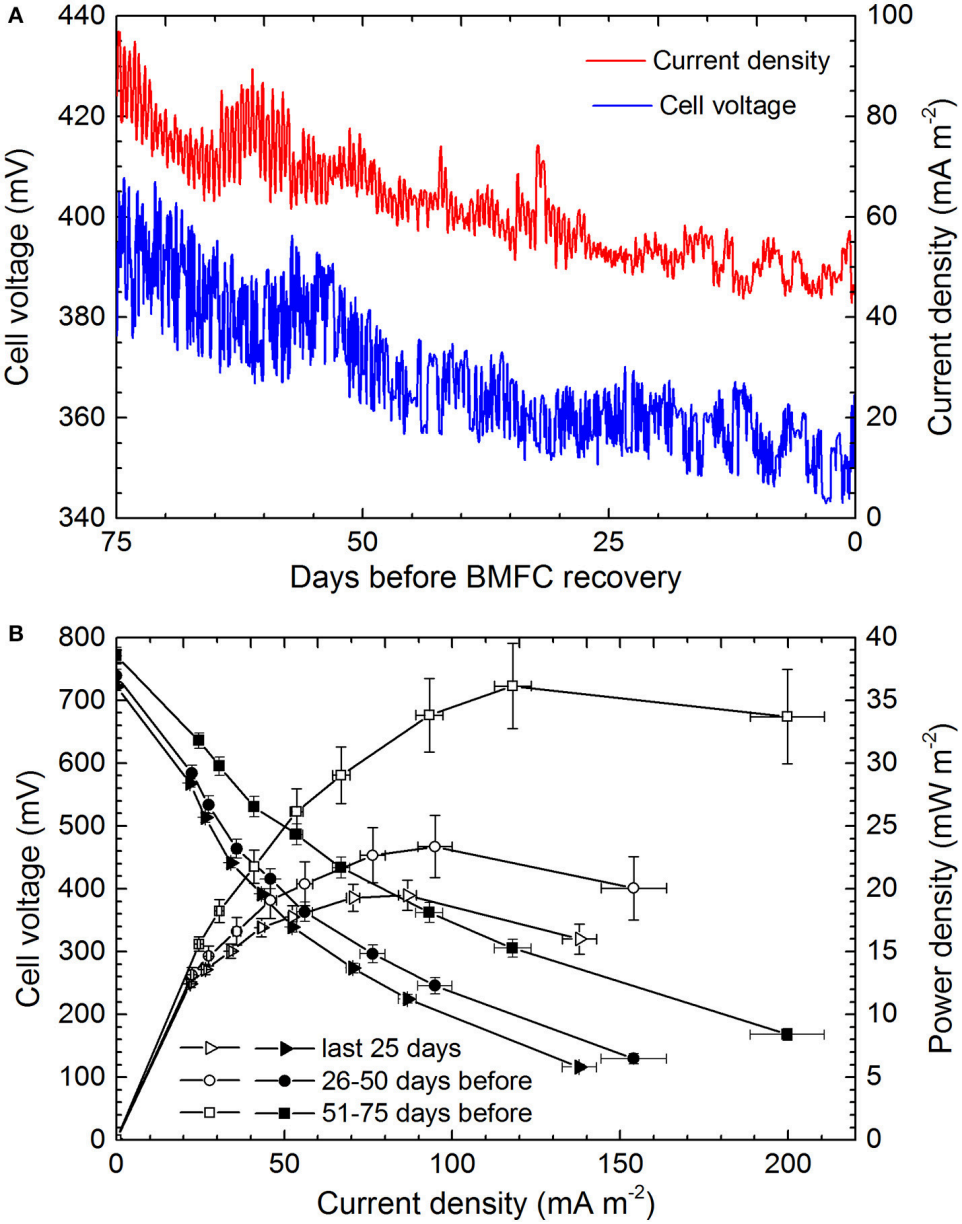
BMFC performance over the 75 days that proceeded recovery and discovery of cable bacteria attached to anode carbon fibers. **(A)** Hourly readings of cell voltage (cathode vs. anode) and current density. **(B)** Comparative 25-day averages of daily polarization (closed symbols) and power curves (open symbols).

To understand the conditions that led to the attachment of filamentous cable bacteria to carbon fiber anodes in the marine environment, we focus first on the cell potentials and current drawn from the BMFC anode over the last 75 days of the deployment (Figure 3). Since energy was being demanded continuously from the BMFC, its operating voltage averaged 368 ± 14 mV and current was drawn at 62 ± 11 mA ${\text{m}}_{\text{seafloor}.}^{-2}$ Both parameters displayed a gradual decline (Figure 3A), indicating the BMFC's overall ability to deliver power was decreasing; this decline was also reflected in daily polarizations and power curves (Figure 3B).

Previous studies have shown that when a BMFC draws power the difference between the operating potential and the open circuit potential arises dominantly from an overpotential of the anode created by mass transport limitations or concentration losses of electron donors (Logan et al., 2006; Reimers et al., 2006). Assuming an average cathode potential in oxic seawater of 300–360 mV vs. Ag/AgCl (as routinely observed in previous experiments; Ryckelynck et al., 2005; Reimers et al., 2006), the anode potential was ~ −80 to 0 mV vs. Ag/AgCl (or ~170 to 250 mV vs. SHE) over the last 75 days. It is this potential range that is inferred to have had the greatest influence on the community composition of the anode's biofilm and to have prompted cable bacteria attachment and electron transfer through extracellular conductive structures and/or mediators (see below). The daily polarizations did however cycle the anode potential over a broader range, and peak power generally occurred at corresponding cell potentials of 225–360 mV (Figure 3B).

The daily return to open circuit potentials >700 mV (Figure 3B) is certain evidence that the chemical environment within the anode chamber was completely and continuously anoxic. The enclosed sediment layer that had been enriched with dried plankton was composed of 20 ± 1 wt.% organic C and 3.8 ± 0.2 total *N* (*n* = 5) initially, but contained only 3.0 ± 1.9 wt. % organic C and 0.29 ± 0.16 wt. % *N* (*n* = 4) at the time of recovery, indicating significant organic matter remineralization. The black color of the sediment and black deposits on the anode chamber wall (Figure 2C) were indicators of iron sulfide precipitation in a sulfidic environment created by sulfate reduction. Pore water samples from the inner sediment smelled faintly of sulfide and when analyzed for nutrients revealed extremely high ammonia concentrations (Table 1) and a pH(NBS) = 6.61. These chemical characteristics are hallmarks of extensive sulfate reduction in a semi-closed system containing sources of iron (Gardner, 1973; Burdige, 2006). Thus, we infer that dissolved sulfide, ammonia, a heterogeneous collection of dissolved organic compounds, and iron sulfide minerals were available as electron donors for microorganisms within the anode chamber.

**Table 1:** Pore water chemistry of internal sediment samples (*n* = 6).

**Pore water analyte**	**Concentration (μM)**
Ammonia	24, 500 ± 4, 900
Nitrate + Nitrite	22 ± 12^*^
Phosphate	315 ± 169
Silicate	515 ± 25

* *Possibly an artifact of ammonia oxidation*.

### SEM

**Figure 4:**
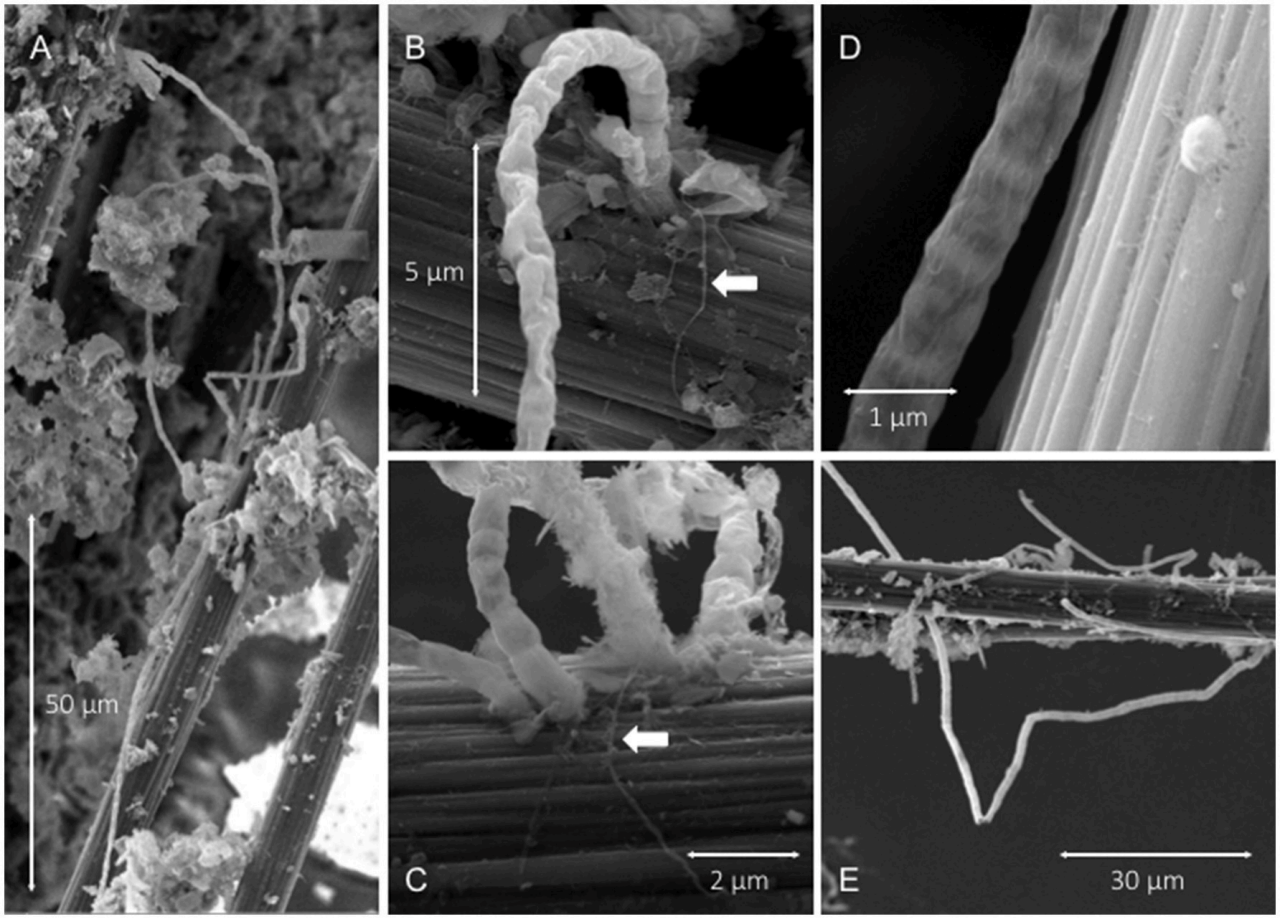
SEM images illustrating: **(A,E)** filamentous cable bacteria attached to carbon fibers amid other aggregated biofilm deposits, **(B,C)** cables attached to carbon fiber surfaces with arrows pointing to radiating pilus-like nano-filamentous structures, **(D)** cell-to-cell boundaries and ridge topography of a cable and an adjacent single cell attached to carbon fiber.

**Figure 5:**
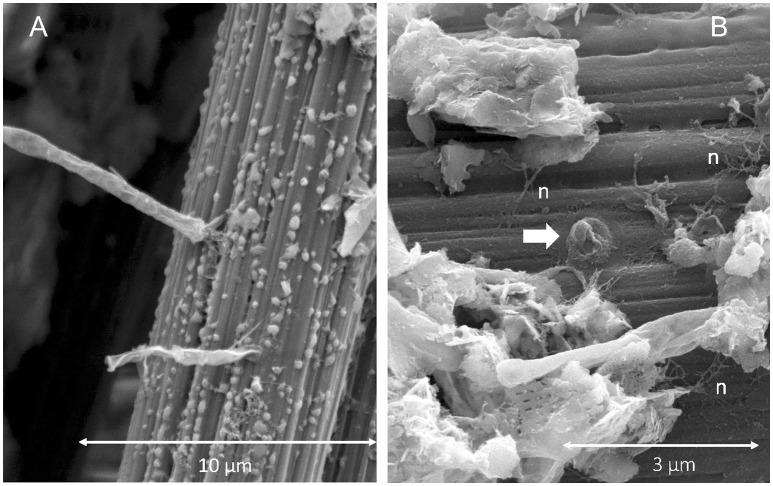
SEM images illustrating: **(A)** mineral deposits adjacent to cable bacteria attachment locations, and **(B)** nano-filament networks (n) across a carbon fiber with arrow pointing to the remnant of a cable attachment.

SEM provided the most striking observations made in this study. As illustrated in Figure 4, cable bacteria filaments with lengths that exceeded 100 μm were observed firmly attached to anode carbon fibers. Attachment was always at the end of a filament with nano-filaments (diameters <100 nm) radiating out from the terminal cell, across the anode fiber surface and sometimes between the attachment locations of adjacent filaments (Figures 4B,C). The dimensions of the linked cells within the cables were ~1.5–2 μm long and generally ~0.8 μm in diameter. Some variation was caused by the SEM-preparatory dehydration process which appeared to collapse or separate outer-membrane materials and to break filaments. Characteristic indentations at cell-cell boundaries and ridge topography (Pfeffer et al., 2012) were visible (Figure 4D). The surfaces of anode fibers also revealed remnant filament attachment “scars,” nano-filament networks or films, isolated bacterial cells, and deposits of amorphous materials and aggregated sediment detritus near bacterial cables (Figure 5). EDS elemental spectra indicated enrichments of S, Si, and Al in the deposits (e.g., deposits in Figure 5A). Not all anode fiber clusters scanned displayed concentrations of cable bacteria filaments, but at least 6 of 10 samples from locations separated over the 4-m length of the anode wire brush had occurrences. The fibers without occurrences appeared to be relatively clean and devoid of a developed biofilm.

### CARD-FISH identification and microbial community analysis

**Figure 6:**
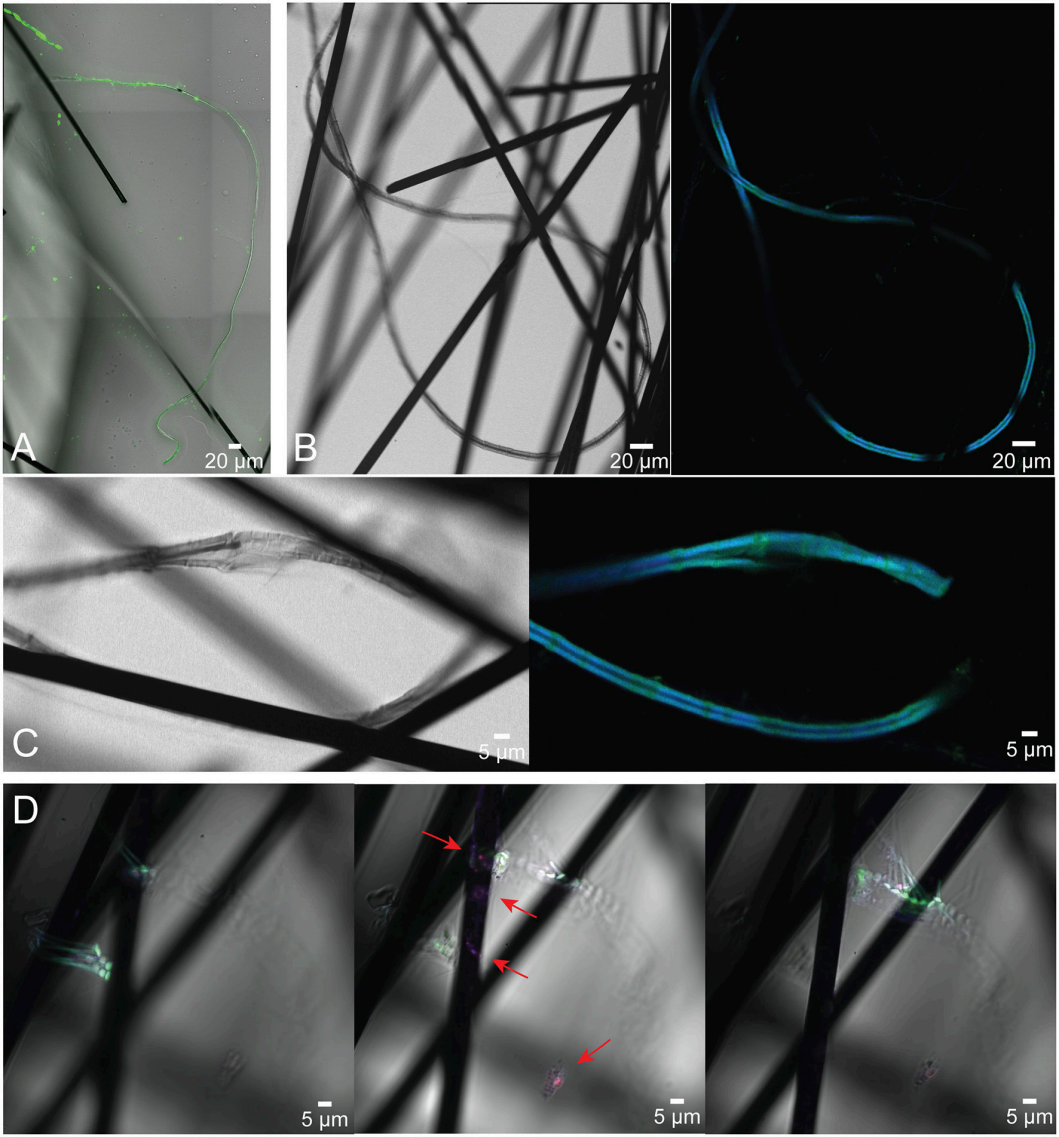
Confocal microscope images of cable bacteria filaments belonging to *Desulfobulbaceae* anchored on anode carbon fibers from the BMFC deployed in Yaquina Bay. Cells were visualized using CARD-FISH (DSB 706 probe + Alexa Fluor 488, green; DRM 432 + Alexa Fluor 555, red; and DSB DAPI, blue). **(A)** Multi-filament bundle visualized with just the green DSB probe; **(B,C)** show long sheathed microbial cables under both transmitted and fluorescent light with both DSB probe and DAPI; in **(D)** the localization of different microbial taxa on the carbon fibers is demonstrated in images taken at varied focal planes (panels from left to right represent the same image frame with different focuses from far to close relative to the microscopic lens; red arrows indicate the location of *Desulfuromonadales*).

CARD-FISH with a *Desulfobulbaceae*-specific oligonucleotide probe confirmed that the microbial filaments anchored on anode fibers belong to the family of *Desulfobulbaceae* and revealed many long fully intact filaments that were well preserved (Figure 6). Under the confocal microscope, probe-hybridized microbial cables exhibited what appeared to be sheathed bundles of filaments that had not been recognized by SEM. The diameters of the inner chains of cells ranged from 0.9 to 1.5 μm, similar to SEM observations and previously reported diameters of microbial cables. However, the diameters of bundles were 5–8 μm, and they appeared to contain up to three filaments (Figure 6C). The longest bundle of filaments observed was 1,365 μm in length. Noteably, clear cell-cell junctions were not always observed in DSB 706 hybridized filaments, possibly because of the complexity of the microscopic visualization around anode fibers, or degradation of the outer membranes of the cable bacteria due to freeze-thaw cycling.

Application of an additional *Desulfuromonadales*-specific oligonucleotide probe (DRM432) helped to confirm concentrations of these known electrogenic bacteria on the carbon fibers surrounding points of cable bacteria attachment (Figure 6D). This probe was selected based on the outcome of the microbial community characterizations described below.

Sequencing of the 16S rRNA gene in DNA extracted from three sections of the anode, and sediment samples inside and outside the BMFC, yielded 5,064 OTUs representing 98 bacterial classes and 7 archaeal classes (OTU table in Datasheet 1; taxonomic assignments in Datasheet 2). One hundred and thirty-six OTUs were assigned to the family *Desulfobulbaceae*. These *Desulfobulbaceae* OTUs do not cluster closely with putative cable bacteria sequences from either marine or freshwater sediments (Schauer et al., 2014; Trojan et al., 2016) or with other *Desulfobulbaceae* phylotypes that have been identified in anode biofilms of waste water MFCs (Ishii et al., 2013b, 2015; see phylogenetic tree in Figure S3). A small subset of sequences from Yaquina Bay anode samples reported by Holmes et al. (2004) as “uncultured deltaproteobacterial clone” were sister to the OTUs in this study but still distinct from known cable bacteria sequences (data not shown).

**Figure 7:**
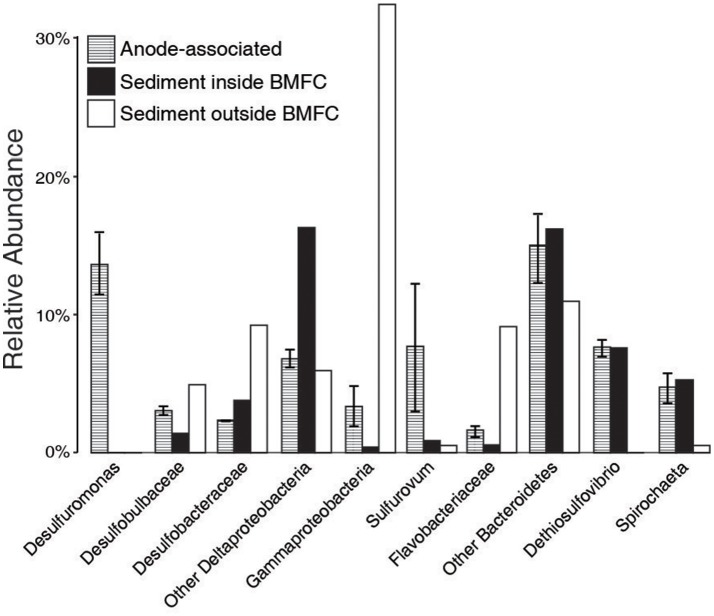
Relative abundance of microbial taxa at multiple taxonomic levels. Error bars indicate standard error across the three anode samples.

Total microbial community composition differed significantly between anode-associated communities within the BMFC and sediments from inside or outside the BMFC (Permanova df = 2, pseudo-*F* = 7.34, *p* = 0.03; Figure 7). Members of the genera *Desulfuromonas* and *Sulfurovum* were highly enriched in the anode-associated communities and contributed 15.9% of the observed dissimilarity to communities inhabiting sediments inside the BMFC and 10.3% to those inhabiting sediments outside the BMFC (similarity percentages analysis). In contrast, *Desulfobulbaceae* contributed <2% to the observed community dissimilarity, and were slightly higher in relative abundance in sediments outside the BMFC than in anode-associated communities. Communities inside the BMFC were also depleted in members of the class *Gammaproteobacteria* compared to sediments outside the BMFC. Microbial community diversity (Shannon-Weiner index; Magurran, 1988) was higher among communities inhabiting sediments outside the BMFC compared to those inside the BMFC or associated with the anode, although this difference was not statistically significant (ANOVA df = 1, *F* = 1.62, *p* = 0.29).

## Discussion

**Figure 8:**
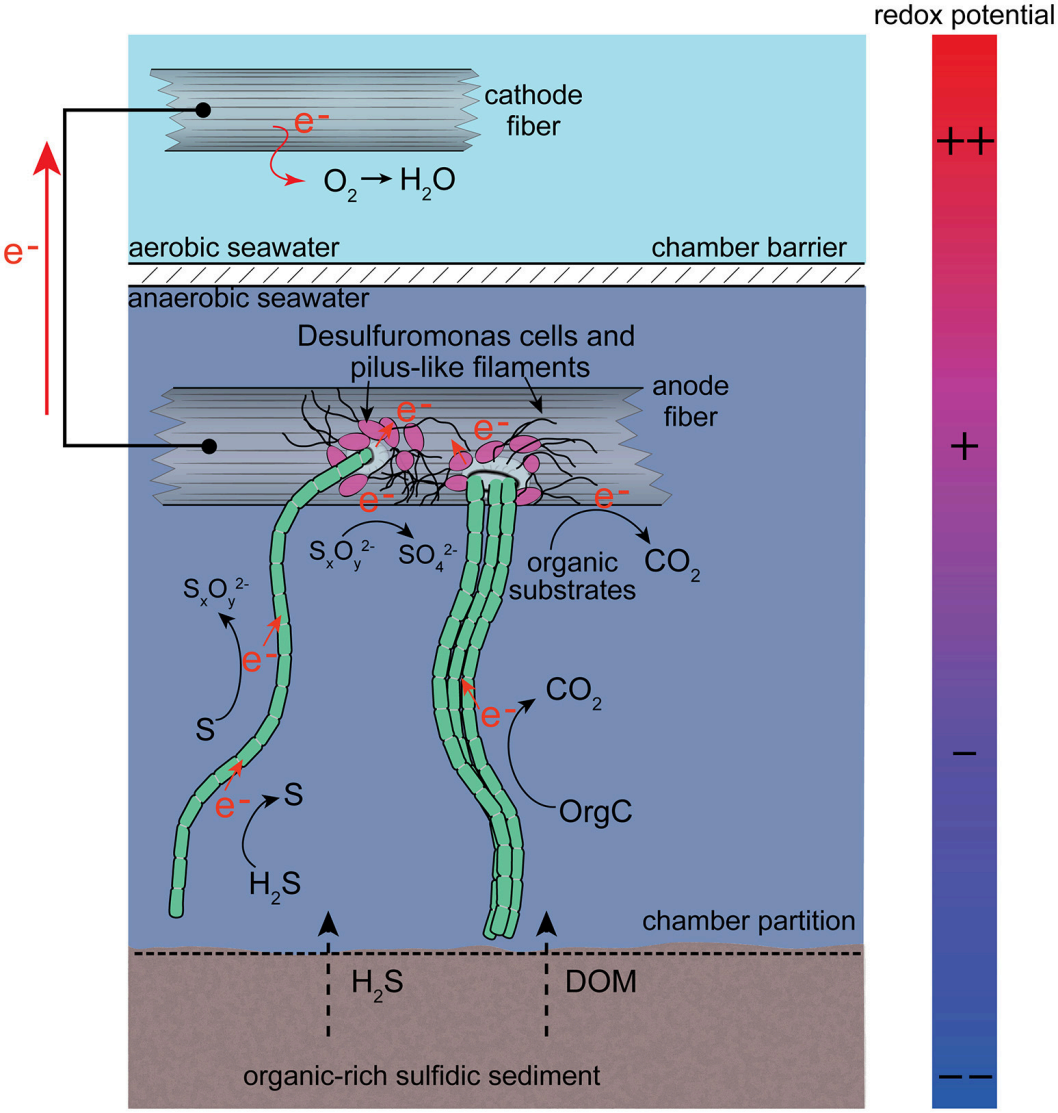
Schematic image of the conditions, microbial associations and processes hypothesized to have contributed to cable bacteria attachment to anode fibers. Electrochemical processes are shown simply rather than as balanced half-cell reactions. The full complement of electron donors and acceptors is not yet known, or where along filaments or bundles of filaments electrons may be harvested. DOM abbreviates dissolved organic matter. Org-C abbreviates organic carbon.

The discovery of long-distance electron transport mediated by filamentous cable bacteria is rapidly changing views of what biological and chemical factors promote redox reactions in aqueous sediments (Vasquez-Cardenas et al., 2015). Until now, models of electrogenic sulfur oxidation by cable bacteria have suggested that access to dissolved O_2_ or at least ${\text{NO}}_{3}^{-}$ was required for the maintainance of their overall metabolic activity (Marzocchi et al., 2014; Meysman et al., 2015; Matturro et al., 2017). The findings of this study indicate that cable bacteria may in fact be facultative anaerobes. When a BMFC is operated with its anode enclosed in an anoxic environment above (or within) anoxic marine sediments, the anode becomes a locus for oxidation reactions that establish a redox gradient and deplete sulfide and other reductants near the anode's surface (Tender et al., 2002; Ryckelynck et al., 2005; Reimers et al., 2013). Furthermore, even though the environment is totally anaerobic the anode's potential shifts toward positive values, and this shift persists even when connection to the cathode is interrupted due to the slow replenishment of reductants by diffusion (Reimers et al., 2013). A migratory and tactile response of cable bacteria to the chemical and potential gradients produced by the anode is illustrated by their mode of attachment that anchors one end of a filament to a carbon fiber surface of the anode. The cables appear able to use a suspended electrode as an electron acceptor and to harvest electrons from chemical donors [e.g., H_2_S and possibly other reductants including dissolved organic matter (DOM), (Nielsen and Risgaard-Petersen, 2015)] diffusing from underlying sediments within the anode chamber (Figure 8). The presence of bundled filaments suggests that cable bacteria may possess more similarities to the “big sulfur bacteria” *Beggiatoa* and *Thioploca* (Jørgensen, 2010) than suggested previously.

The current densities of BMFC when normalized to seafloor area (as in Figure 3) match current densities estimated from rates of oxygen consumption in sediments with cable bacteria, e.g., ~10–100 mA m^−2^ (Nielsen et al., 2010; Malkin and Meysman, 2015). Neither estimate implies all electrons flow solely through microbial cables but rather that an electrogenic microbiological network links electron donors with an available electron sink (oxygen or electrode). The likelihood that microbes “hook up” together to form conductive, multi-species aggregates and biofilms (Ishii et al., 2015; Vasquez-Cardenas et al., 2015; Lovley, 2017) is further supported by clusters of individual cells, nano-filaments and attached cable bacteria bundles as detected by SEM and CARD-FISH. The multiprobe CARD-FISH highlighted that many of the individual cells were from the family *Desulfuromonadaceae*. However, the end attachment of cables to anode fibers also strongly suggests the cable bacteria can independently perform extracellular electron transport to solid substrates when soluble electron acceptors such as oxygen are not available.

The contributions of cable bacteria to microbial communities associated with electrodes harvesting electricity from aquatic sediments or waste water may have been inadvertently missed in past studies because their taxonomy has only been described recently and their outer cell structures may hamper cell lysis and DNA extraction (Trojan et al., 2016). After seeing cables stand out predominantly under SEM and by CARD-FISH, we were surprised *Desulfobulbaceae* represented <5% of the 16S OTUs associated with anode samples and that their relative abundance was not enriched compared to the outer sediment sample from the deployment location (Figure 7). Phylogenetic analysis of the *Desulfobulbaceae* OTUs that were recovered from the anode found that these OTUs are distantly related to previously identified cable bacteria, but cluster with other *Desulfobulbaceae* previously found to be highly enriched on a prototype BMFC anode that had been deployed in Yaquina Bay (Holmes et al., 2004). This suggests that: (1) cable bacteria may have a broader taxonomic diversity within the family *Desulfobulbaceae* than previously appreciated, and (2) relative abundance estimates of cable bacteria are especially sensitive to DNA-extraction methodologies.

The earlier BMFC experiment in Yaquina Bay used a graphite plate anode buried within the sediments rather than a carbon fiber brush in a chamber, and it applied more aggressive DNA extraction and clean-up methods than used in this study. Holmes et al. (2004) and Ryckelynck et al. (2005) speculated the enrichment of *Desulfobulbus/Desulfocapsa* was related to a general ability of these bacteria to anaerobically oxidize S° (that collects due to chemical oxidation on the anode) to sulfate with the electrode as electron acceptor or else to recycle S° by a disproportionation reaction. Many of the members of the microbial community within the anodic chamber of a marine BMFC appear likely to take advantage of multiple-step oxidations from sulfide to S^0^, ${\text{S}}_{\text{x}}^{2-}$, S_4_${\text{O}}_{6}^{2-}$, S_2_${\text{O}}_{3}^{2-}$, and eventually ${\text{SO}}_{4}^{2-}$ including *Sulfurovum* and *Dethiosulfovibrio* (Figure 7). The extent of these sulfur transformations should depend on the anode potential, and be accelerated by microbial catalysis utilizing the electrode as the terminal electron acceptor (Schippers, 2004; Sun et al., 2009; Gong et al., 2013). Cable bacteria filaments may be uniquely able to accept electrons from different sulfur intermediates (as well as other electron donors) at different points along chains of cells, as they both produce and further oxidize sulfur intermediates (Figure 8). It is noteworthy that this hypothetical metabolic framework differs from the microbial community network depicted by Matturro et al. (2017) for an engineered system for the treatment of contaminated sediments, called the “oil spill snorkel.” The snorkel consists of a single graphite rod penetrating oxic and anoxic sediments and serving as both anode and cathode. In this electrode configuration, cable bacteria were proposed to perform sulfide oxidation in parallel with the inserted graphite rod; they were not proposed to attach to the electrode (Matturro et al., 2017).

The attachment mechanism between cable bacteria and anodes of BMFCs appears to involve only single cells at the ends of filaments. Sheath and nano-filament structures observed at the anchor site of filaments may be produced by the cable bacteria, but a recent genomic study by Holmes et al. (2016) suggests it is more likely that if the nano-filaments have the properties of conductive pili, they were produced by bacteria from within the order Desulfuromonadales. As *Desulfuromonas* in the family *Desulfuromonadaceae* belonging to the order Desulfuromonadales were enriched on anode fiber samples (Figure 7), they are candidates for the observed nano-filament production and for forming consortia with cable bacteria.

Lastly, as the conduction mechanism of cable bacteria has not been verified (Lovley, 2017), the electrochemical properties of the attachment materials merit future research. It is unclear if the observed films and nano-filaments help with anchoring, with electron transfer, or both functions. Previous studies using waste water-fed and clarifier effluent-fed MFCs suggested that certain *Desulfobulbaceae* respond to surface potential changes by gene expression of *c*-type cytochromes functioning as redox-active components of outer membranes (Ishii et al., 2013a,b). Mediators of intermediate formal potentials contained in outer cell membranes or in an extracellular biofilm matrix, including cytochrome complexes, may be key contributors to electron transfer at the point of electrode attachment (as has been proposed for other biofilms and microbial consortia, McGlynn et al., 2015; and indicated by additional metatranscriptomics, Ishii et al., 2015). Ionic charge carriers in the surrounding seawater solution contribute a charge balance for electrons passed from anode to cathode, and shifts in anode potential (especially during daily BMFC polarizations) likely cause the redox mediators to have periods of being predominantly reduced by the cells and then reoxidized by the anode. Such potential cycling has been shown to be beneficial to BMFC performance while having little impact on microbial diversity (Gardel et al., 2012).

## Future research

Our results add to the growing body of knowledge about cable bacteria metabolism by showing that these bacterial filaments can attach to positively-poised electrodes and by suggesting they can use an electrode as an electron acceptor. Many questions for future research remain. Most notably: (1) what is the full spectrum of electron donors utilizable by cable bacteria and where along a filament may electrons be harvested from external donors; (2) what is the mechanism of cell-to-cell electron conduction from end-to-end of a cable; (3) what causes the filaments to form bundles; (4) how do cable bacteria interact with other bacteria capable of extracellular electron transfer; (5) how do they attach to a solid electron acceptor; (6) do they produce conductive pili or form connections to pili produced by other microrganisms; (7) how genetically diverse are cable bacteria; (8) are all cable bacteria facultative anaerobes; and (9) will the enrichment of cable bacteria in microbial communities on a BMFC anode enhance the device's electrical energy recovery.

## Author contributions

CR: Primary author of the paper, designed field experiment and follow-up analyses, recognized the discovery. CL: Performed CARD-FISH and wrote paper sections on this method and results. MG: Did phylogenetic analysis, prepared figures, and contributed to writing. PS: Maintained field experiment and collected samples. MW: Developed the power management system that maintained the microbial fuel cell and collected data on its performance.

### Conflict of interest statement

The authors declare that the research was conducted in the absence of any commercial or financial relationships that could be construed as a potential conflict of interest. The reviewer KN and handling Editor declared their shared affiliation.

## References

[B1] Berner R. (1963). Electrode studies of hydrogen sulfide in marine sediments. Geochim. Cosmochim. Acta 27, 563–575. 10.1016/0016-7037(63)90013-9

[B2] Brown C. Ozretich R. (2009). Coupling between the coastal ocean and Yaquina Bay, Oregon: importance of oceanic inputs relative to other nitrogen sources. Estuar. Coasts 32, 219–237. 10.1007/s12237-008-9128-6

[B3] Burdige D. (2006). Geochemistry of Marine Sediments. Princeton, NJ: Princeton University Press.

[B4] Gardel E. Nielsen M. Grisdela P. J. Girguis P. (2012). Duty cycling influences current generation in multi-anode environmental microbial fuel cells. Environ. Sci. Technol. 46, 5222–5229. 10.1021/es204622m 22497491

[B5] Gardner L. (1973). Chemical models for sulfate reduction in closed anaerobic marine environments. Geochim. Cosmochim. Acta 37, 53–68. 10.1016/0016-7037(73)90243-3

[B6] Gong Y. Ebrahim A. Feist A. Embree M. Zhang T. Lovley D.. (2013). Sulfide-driven microbial electrosynthesis. Environ. Sci. Technol. 47, 568–573. 10.1021/es303837j 23252645

[B7] Gong Y. Radachowsky S. Wolf M. Nielsen M. Girguis P. Reimers C. (2011). Benthic microbial fuel cell as direct power source for an acoustic modem and seawater oxygen/temperature sensor system. Environ. Sci. Technol. 45, 5047–5053. 10.1021/es104383q 21545151

[B8] Gorby Y. Yanina S. McLean J. Rosso K. Moyles D. Dohnalkova A.. (2006). Electrically conductive bacterial nanowires produced by *Shewanella* oneidensis strain MR-1 and other microorganisms. Proc. Natl. Acad. Sci. U.S.A. 103, 11358–11363. 10.1073/pnas.0604517103 16849424 PMC1544091

[B9] Gorley R. Clarke K. (2006). PRIMER V6: User Manual-Tutorial. Plymouth Marine Laboratory.

[B10] Holmes D. Bond D. O'Neil R. Reimers C. Tender L. Lovley D. (2004). Microbial communities associated with electrodes harvesting electricity from a variety of aquatic sediments. Microb. Ecol. 48, 178–190. 10.1007/s00248-003-0004-4 15546038

[B11] Holmes D. Dang Y. Walker D. Lovley D. (2016). The electrically conductive pili of geobacter species are a recently evolved feature for extracellular electron transfer. Microb. Genom. 2:e000072. 10.1099/mgen.0.000072 28348867 PMC5320591

[B12] Ishii S. Shimoyama T. H. Watanabe K. (2008). Characterization of a filamentous biofilm community established in a cellulose-fed microbial fuel cell. BMC Microbiol. 8:6. 10.1186/1471-2180-8-6 18186940 PMC2254626

[B13] Ishii S. Suzuki S. Norden-Krichmar T. Tenney A. Chain P. Scholz M.. (2013a). A novel metatranscriptomic approach to identify gene expression dynamics during extracellular electron transfer. Nat. Commun. 4, 1601. 10.1038/ncomms2615 23511466

[B14] Ishii S. Suzuki S. Norden-Krichmar T. Wu A. Yamanaka Y. Nealson K.. (2013b). Identifying the microbial comunities and operational conditions for optimized wastewater treatment in microbial fuel cells. Water Res. 47, 7120–7130. 10.1016/j.watres.2013.07.048 24183402

[B15] Ishii S. Suzuki S. Tenney A. Norden-Krichmar T. Nealson K. Bretschger O. (2015). Microbial metabolic networks in a complex electrogenic biofilm recovered from a stimulus-induced metatranscriptomics approach. Sci. Rep. 5:14840. 10.1038/srep14840 26443302 PMC4595844

[B16] Jørgensen B. (2010). Big sulfur bacteria. ISME J. 4, 1083–1084. 10.1038/ismej.2010.106 20631811

[B17] Jørgensen B. Kasten S. (2006). Sulfur cycling and methane oxidation, in Marine Geochemistry, eds Schulz H. Zabel M. (Berlin: Springer), 271–310.

[B18] Kato S. Hashimoto K. Wantanabe K. (2012). Microbial interspecies electron transfer via electric currents through conductive minerals. Proc. Natl. Acad. Sci. U.S.A. 109, 10042–10046. 10.1073/pnas.1117592109 22665802 PMC3382511

[B19] Kozich J. Westcott S. Baxter N. Highlander S. Schloss P. (2013). Development of a dual-index sequencing strategy and curation pipeline for analyzing amplicon sequence data on the MiSeq Illumina sequencing platform. Appl. Environ. Microbiol. 79, 5112–5120. 10.1128/AEM.01043-13 23793624 PMC3753973

[B20] Li C. Lesnik K. Fan Y. Liu H. (2016). Millimeter scale electron conduction through exoelectrogenic mixed species biofilms. FEMS Microbiol. Lett. 363:fnw153. 10.1093/femsle/fnw153 27279626 PMC5975238

[B21] Logan B. Hamelers B. Rozendal R. Schröder U. Keller J. Freguia S.. (2006). Microbial fuel cells: methodology and technology. Environ. Sci. Technol. 40, 5181–5192. 10.1021/es0605016 16999087

[B22] Lovley D. (2012). Electromicrobiology. Annu. Rev. Microbiol. 66, 391–409. 10.1146/annurev-micro-092611-150104 22746334

[B23] Lovley D. (2017). Happy together: microbial communities that hook up to swap electrons. ISME J. 11, 327–336. 10.1038/ismej.2016.136 27801905 PMC5270577

[B24] Lovley D. Ueki T. Zhang T. Malvankar N. Shretha P. Flanagan K.. (2011). Geobacter: the microbe electric's physiology, ecology, and practical applications. Adv. Microb. Physiol. 59, 1–100. 10.1016/B978-0-12-387661-4.00004-5 22114840

[B25] Lücker S. Steger D. Kjeldsen K. Macgregor B. (2007). Improved 16S rRNA-targeted probe set for analysis of sulfate-reducing bacteria by fluorescence *in situ* hybridization. J. Microbiol. Methods 69, 523–528. 10.1016/j.mimet.2007.02.009 17408790

[B26] Magurran A. (1988). Diversity indices and species abundance models, in Ecological Diversity and Its Measurement, ed Magurran A. (Dordrecht: Springer), 7–45. 10.1007/978-94-015-7358-0_2

[B27] Malkin S. Meysman F. (2015). Rapid redox signal transmission by “cable bacteria” beneath a photosynthetic biofilm. Appl. Environ. Microbiol. 81, 948–956. 10.1128/AEM.02682-14 25416774 PMC4292484

[B28] Malkin S. Rao A. Seitaj D. Vasquez-Cardenas D. Zetsche E.-M. Hidalgo-Martinez S.. (2014). Natural occurrence of microbial sulpher oxidation by long-range electron transport in the seafloor. ISME J. 8, 1843–1854. 10.1038/ismej.2014.41 24671086 PMC4139731

[B29] Malvankar N. Tuominen M. Lovley D. (2012). Lack of cytochrome involvement in long-range electron transport through conductive biofilms and nanowires of *Geobacter sulfurreducens*. Energy Environ. Sci. 5, 8651–8659. 10.1039/c2ee22330a

[B30] Malvankar N. Yalcin S. Tuominen M. Lovley D. (2014). Visualization of charge propagation along individual pili proteins using ambient electrostatic force microscopy. Nat. Nonotechnol. 9, 1012–1017. 10.1038/nnano.2014.236 25326694

[B31] Marsili E. Baron D. Shikhare I. Coursolle D. Gralnick J. Bond D. (2008). Shewanella secretes flavins that mediate extracellular electron transfer. Proc. Natl. Acad. Sci. U.S.A. 105, 3968–3973. 10.1073/pnas.0710525105 18316736 PMC2268775

[B32] Marzocchi U. Trojan D. Larsen S. Meyer L. Revsbech N. Schramm A.. (2014). Electric coupling between distant nitrate reduction and sulfide oxidation in marine sediment. ISME J. 8, 1682–1690. 10.1038/ismej.2014.19 24577351 PMC4817607

[B33] Matturro B. Viggi C. C. Aulenta F. Rossetti S. (2017). Cable bacteria and the bioelectrochemical snorkel: the natural and engineered facets playing a role in hydrocarbons degradation in marine sediments. Front. Microbiol. 8:952. 10.3389/fmicb.2017.00952 28611751 PMC5447156

[B34] McGlynn S. Chadwick G. Kempes C. Orphan V. (2015). Single cell activity reveals direct electron transfer in methanotrophic consortia. Nature 526, 531–535. 10.1038/nature15512 26375009

[B35] Meysman F. Risgaard-Petersen N. Malkin S. Nielsen L. (2015). The geochemical fingerprint of microbial long-distance electron transport in the seafloor. Geochim. Cosmochim. Acta 152, 122–142. 10.1016/j.gca.2014.12.014

[B36] Nevin K. Kim B. Glaven R. Johnson J. Woodard T. Methe B.. (2009). Anode biofilm transcriptomics reveals outer surface components essential for high density current production in *Geobacter* sulfurreducens fuel cells. PLoS ONE 4:e5628. 10.1371/journal.pone.0005628 19461962 PMC2680965

[B37] Nielsen L. Risgaard-Petersen N. (2015). Rethinking sediment biogeochemistry after the discovery of electric currents. Annu. Rev. Mar. Sci. 7, 424–442. 10.1146/annurev-marine-010814-015708 25251266

[B38] Nielsen L. Risgaard-Petersen N. Fossing H. Christensen P. Sayama M. (2010). Electric currents couple spatially separated biogeochemical processes in marine sediment. Nature 463, 1071–1074. 10.1038/nature08790 20182510

[B39] Nielsen M. Reimers C. Stecher H. I. (2007). Enhanced power from chambered benthic microbial fuel cells. Environ. Sci. Technol. 41, 7895–7900. 10.1021/es071740b 18075105

[B40] Nielsen M. Reimers C. White H. Sharma S. Girguis P. (2008). Sustainable energy from deep ocean cold seeps. Energy Environ. Sci. 1, 584–593. 10.1039/b811899j

[B41] Oksanen J. Blanchet F. G. Kindt R. Legendre P. Minchin P. R. O'Hara R. B. (2013). Package ‘vegan.’ Community Ecology Package, Version 2.4-4.

[B42] Pernthaler A. Pernthaler J. Amann R. (2002). Fluorescence *in situ* hybridization and catalyzed reporter deposition for the identification of marine bacteria. Appl. Environ. Microbiol. 68, 3094–3101. 10.1128/AEM.68.6.3094-3101.2002 12039771 PMC123953

[B43] Pfeffer C. Larsen S. Song J. Dong M. Besenbacher F. Meyer R.. (2012). Filamentous bacteria transport electrons over centimetre distances. Nature 491, 218–221. 10.1038/nature11586 23103872

[B44] Pirbadian S. Barchinger S. Leung K. Byun H. Jangir Y. Bouhenni R.. (2014). Shewanella oneidensis MR-1 nanowires are outer membrane and periplasmic extensions of the extracellular electron transport components. Proc. Natl. Acad. Sci. U.S.A. 111, 12881–12888. 10.1073/pnas.1410551111 25143589 PMC4156777

[B45] Reimers C. (2015). Applications of bioelectrochemical energy harvesting in the marine environment, in Electrochemically Active Biofilms in Microbial Fuel Cells and Bioelectrochemical Systems: From Laboratory Practice to Data Interpretation, eds Beyenal H. Babauta J. (Hoboken, NJ: Elsevier), 345–366.

[B46] Reimers C. Alleau Y. Bauer J. Delaney J. Girguis P. Schrader P. (2013). Redox effects on microbial degradation of refractory organic matter in marine sediments. Geochim. Cosmochim. Acta 121, 582–598. 10.1016/j.gca.2013.08.004

[B47] Reimers C. Girguis P. Stecher H. III. Tender L. Ryckelynck N. Whaling P. (2006). Microbial fuel cell energy from an ocean cold seep. Geobiology 4, 123–136. 10.1111/j.1472-4669.2006.00071.x

[B48] Ryckelynck N. Stecher H. III. Reimers C. (2005). Understanding the anodic mechanism of a seafloor fuel cell: interactions between geochemistry and microbial activity. Biogeochemistry 76, 113–139. 10.1007/s10533-005-2671-3

[B49] Schauer R. Risgaard-Petersen N. Kjeldsen K. Tataru Bjerg J. Jorgensen B. Schramm A.. (2014). Succession of cable bacteria and electric currents in marine sediment. ISME J. 8, 1314–1322. 10.1038/ismej.2013.239 24451206 PMC4030233

[B50] Schippers A. (2004). Biogeochemistry of metal sulfide oxidation in mining environments, sediments, and soils. Geol. Soc. Am. Special Papers 379, 49–62. 10.1130/0-8137-2379-5.49

[B51] Schrader P. Reimers C. Girguis P. Delaney J. Doolan C. Wolf M. (2016). Independent benthic microbial fuel cells powering sensors and acoustic communications with the MARS underwater observatory. J. Atmos. Oceangr. Technol. 33, 607–617. 10.1175/JTECH-D-15-0102.1

[B52] Schröder U. (2007). Anodic electron transfer mechanisms in microbial fuel cells and their energy efficiency. Phys. Chem. Chem. Phys. 9, 2619–2629. 10.1039/B703627M 17627307

[B53] Stumm W. Morgan J. (1981). Aquatic Chemistry An Introduction Emphasizing Chemical Equilibria in Natural Waters, 2nd Edn. New York, NY: John Wiley and Sons.

[B54] Summers Z. Fogarty H. Leang L. Franks A. Malvankar N. Lovley D. (2010). Direct exchange of electrons with aggregates of an evolved syntrophic coculture of anaerobic bacteria. Science 330, 1413–1415. 10.1126/science.1196526 21127257

[B55] Sun M. Mu Z.-X. Chen Y.-P. Sheng G.-P. Liu X.-W. Chen Y.-Z.. (2009). Microbe-assisted sulfide oxidation in the anode of a microbial fuel cell. Environ. Sci. Technol. 43, 3372–3377. 10.1021/es802809m 19534160

[B56] Tender L. Reimers C. Stecher H. III. Holmes D. Bond D. Lowy D. (2002). Harnessing microbial generated power on the seafloor. Nat. Biotechnol. 20, 821–825. 10.1038/nbt716 12091916

[B57] Trojan D. Schreiber L. Bjerg J. Boggild A. Yang T. Kjeldsen K.. (2016). A taxonomic framework for cable bacteria and the proposal of the candidate genera Electrothrix and Electronema. Syst. Appl. Microbiol. 39, 297–306. 10.1016/j.syapm.2016.05.006 27324572 PMC4958695

[B58] Vasquez-Cardenas D. Van de Vossenberg J. Polerecky L. Malkin S. Schauer R. Hidalgo-Martinez S.. (2015). Microbial carbon metabolism associated with electrogenic sulphur oxidation in coastal sediments. ISME J. 9, 1966–1978. 10.1038/ismej.2015.10 25679534 PMC4542026

[B59] Verardo D. J. Froelich P. McIntyre A. (1990). Determination of organic carbon and nitrogen in marine sediments using the Carlo Erba NA-1500 analyzer. Deep-Sea Res. Part A 37, 157–165. 10.1016/0198-0149(90)90034-S

[B60] Wendeberg A. (2010). Fluorescence *in situ* hybridization for the identification of environmental microbes. Cold Spring Harb. Protoc. 2010:pdb.prot5366. 10.1101/pdb.prot5366 20150125

