# Supplementary figures and images for: The Identification of Cable Bacteria Attached to the Anode of a Benthic Microbial Fuel Cell: Evidence of Long Distance Extracellular Electron Transport to Electrodes
Source: Front Microbiol. 2017 Oct 24;8:2055. doi: 10.3389/fmicb.2017.02055 (PMC5660804; doi:10.3389/fmicb.2017.02055)

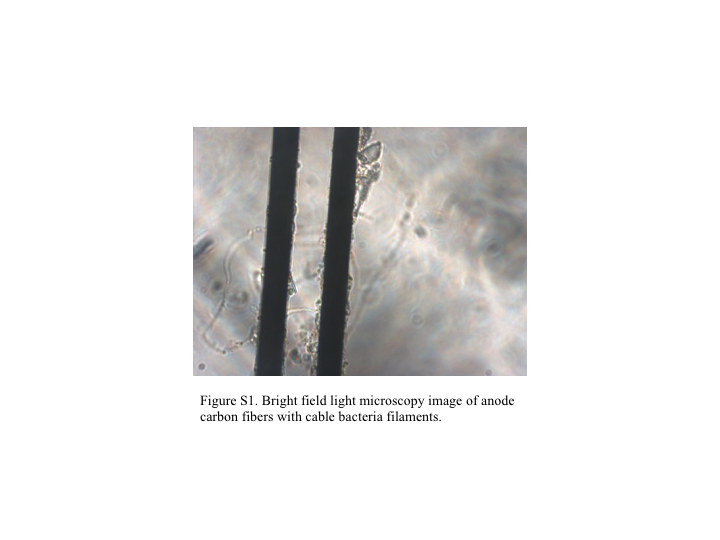

Supplement: Supplementary file 1 [file Image1.TIFF]

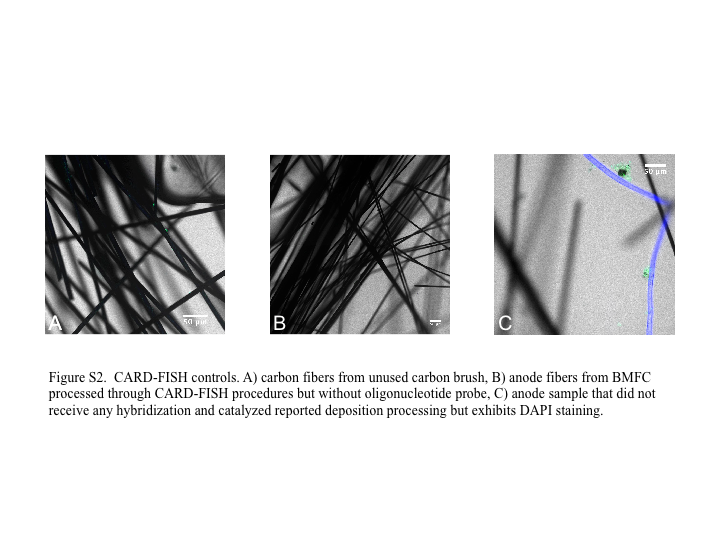

Supplement: Supplementary file 2 [file Image2.TIFF]

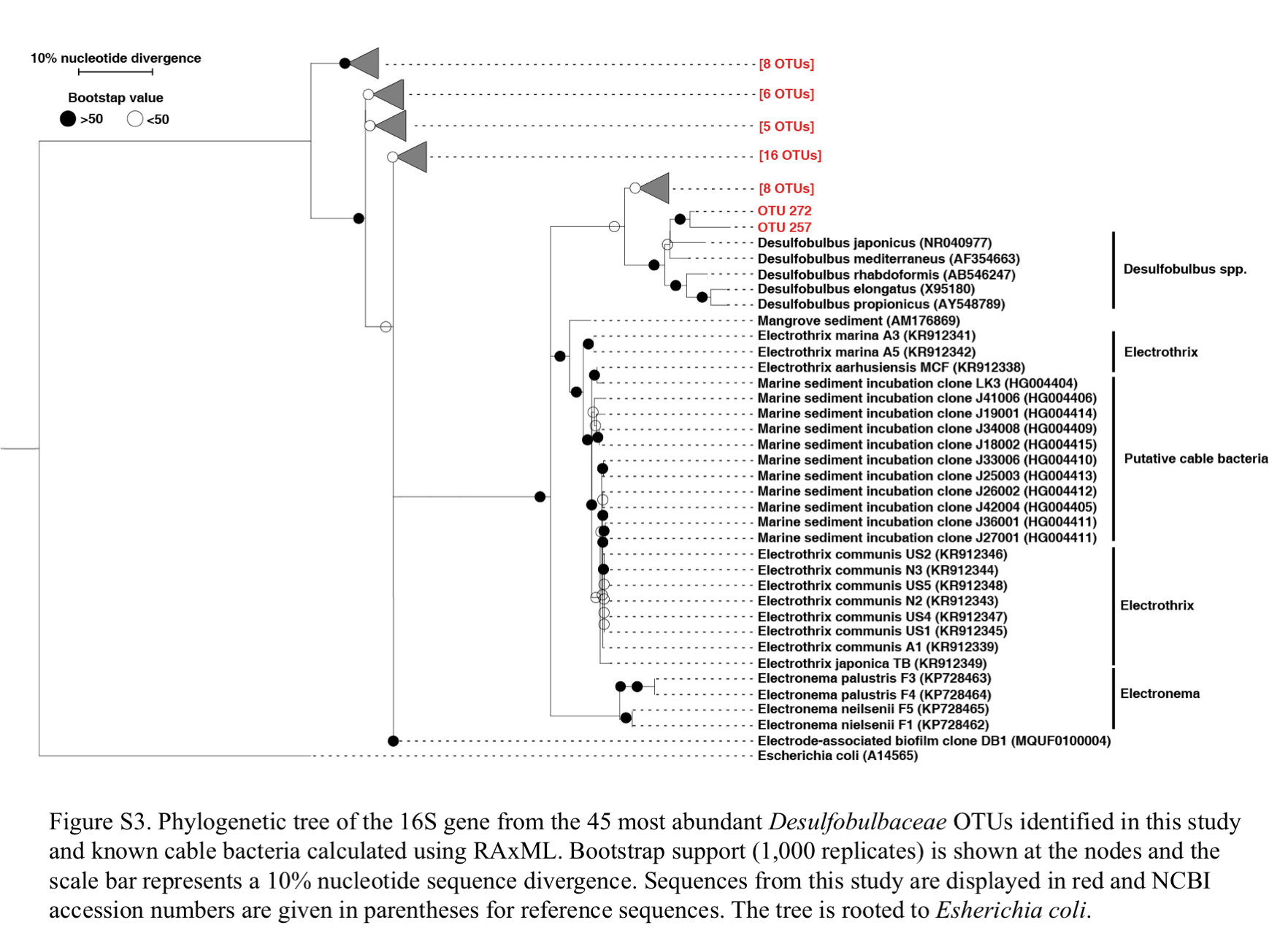

Supplement: Supplementary file 3 [file Image3.TIFF]
